# On the Construction, Etc., Etc., of Hospitals for the Insane

**Published:** 1854-12

**Authors:** 


*On the Construction, Organization, and General Arrangements of Hospitals for the Insane.* By THOMAS S. KIRKBRIDE, M. D., Physician to the Pennsylvania Hospital for the Insane. Philadelphia: Lindsay & Blakiston. 1854.

It is now generally conceded, that a Hospital for the Insane should not contain more than 250 inmates. Accordingly, Dr. Kirkbride has here given us a plan for such a building, and makes that plan the text for many interesting remarks upon construction and arrangements, ventilation and warming

Dr. Kirkbride is admitted to be one of the most accomplished physicians for the insane in this country, (and this country excels in this department of medicine,) and an experience of sixteen years has given him great familiarity with the necessities of such buildings.

But among all the model buildings mentioned by Dr. K., we find no mention of the Insane Department of the Erie Co. Almshouse. We do not wish to impeach Dr. K.'s familiarity with buildings of this class, neither would we impute to him any local jealousies. But assuredly, Dr. Kirkbride *must* have heard of this model institution. Why did he not visit it before writing his book? Had he done so he would have seen the most artistically contrived cells for the confinement of this wicked class, so arranged that no bedding or clothing can possibly be torn by their frantic motions, these articles being prudently removed. Then, too, he would have learned something about straight-jackets and handcuffs. We dare say that Dr. Kirkbride, in all the great asylum under his charge, has not a tithe of these ingenious contrivances, compared to those which are endorsed every year by the Erie County Board of Supervisors as remarkably neat, convenient, and *economical.*

But for all that benighted class of individuals who live outside of the benign influences of our honored home institution, this work of Dr. Kirkbride's will prove very valuable. Indeed we are not sure that a copy of it should not be gratuitously furnished to our county authorities—not by way of instruction, for of that they have no need—but to afford them a pleasant and satisfactory feeling when they contrast its propositions with the perfect system they have the honor of guiding.

For sale by MILLER, ORTON & MULLIGAN. H.

